# An investigation into the effects of excipient particle size, blending techniques and processing parameters on the homogeneity and content uniformity of a blend containing low-dose model drug

**DOI:** 10.1371/journal.pone.0178772

**Published:** 2017-06-13

**Authors:** Hamad Alyami, Eman Dahmash, James Bowen, Afzal R. Mohammed

**Affiliations:** 1 Aston School of Pharmacy, Aston University, Birmingham, United Kingdom; 2 Department of Engineering and Innovation, Open University, Milton Keynes, United Kingdom; Kermanshah University of Medical Sciences, ISLAMIC REPUBLIC OF IRAN

## Abstract

Powder blend homogeneity is a critical attribute in formulation development of low dose and potent active pharmaceutical ingredients (API) yet a complex process with multiple contributing factors. Excipient characteristics play key role in efficient blending process and final product quality. In this work the effect of excipient type and properties, blending technique and processing time on content uniformity was investigated. Powder characteristics for three commonly used excipients (starch, pregelatinised starch and microcrystalline cellulose) were initially explored using laser diffraction particle size analyser, angle of repose for flowability, followed by thorough evaluations of surface topography employing scanning electron microscopy and interferometry. Blend homogeneity was evaluated based on content uniformity analysis of the model API, ergocalciferol, using a validated analytical technique. Flowability of powders were directly related to particle size and shape, while surface topography results revealed the relationship between surface roughness and ability of excipient with high surface roughness to lodge fine API particles within surface groves resulting in superior uniformity of content. Of the two blending techniques, geometric blending confirmed the ability to produce homogeneous blends at low dilution when processed for longer durations, whereas manual ordered blending failed to achieve compendial requirement for content uniformity despite mixing for 32 minutes. Employing the novel dry powder hybrid mixer device, developed at Aston University laboratory, results revealed the superiority of the device and enabled the production of homogenous blend irrespective of excipient type and particle size. Lower dilutions of the API (1% and 0.5% w/w) were examined using non-sieved excipients and the dry powder hybrid mixing device enabled the development of successful blends within compendial requirements and low relative standard deviation.

## Introduction

Oral drug delivery is the favoured route of drug delivery, due to the convenience of administration, being non-invasive and thus more likely to promote patients compliance with their treatments [1]. Efficient and reproducible blending process is critical to manufacturing of oral drug delivery systems, as the quality of the final product is driven by the quality of the blend. Therefore, production of non-homogenous blends results in discrepancy in the content of the active pharmaceutical ingredient (API) and product failure [2].

Blending is a process where two ingredients or more are processed in order to achieve a homogenous product [3, 4]. To achieve that, three main mechanisms of powders blending are involved; convection, diffusion, and shear. Convective blending encompasses gross movement of particles within the blend, whereas, diffusion is a slow blending process where individual particles are distributed upon blending into newly formed interface. Lastly, the shear mechanism of blending comprises of blending material while passing along forced slip planes which could aid in breaking agglomerates and enable blending [4–6]. Depending on the flow characteristics of powders, solids are broadly divided into cohesive materials and non-cohesive materials. Blending of cohesive materials is more complex because of the possibility of developing aggregates and lumps [3, 4].

Normally, high drug loading capability of excipients is ideal in such formulations; the overall weight of tablets is relatively low to allow for rapid disintegration and dissolution [7]. However, for high potency or low load drugs like vitamins that exist at lower loading in formulations, this is a major issue. The problem with manufacturing using such drugs is obtaining a uniform distribution of drug throughout the formulation [8]. Uniformity of API is important as it will impact drug dissolution, absorption, bioavailability and onset of clinical effect [9].

Developing formulation for low load drugs where small amount of the API is blended with a large amount of excipients/ carriers is challenging. Blend homogeneity is dependent on multiple factors including: particle size, size distribution and density of the individual components, blending process or blending equipment and presence of agglomerates within the blend [6, 10]. Understanding of powder properties particularly particle size, shape, size distribution particle surface roughness will enable the selection of appropriate excipients and blending process [2, 11, 12].

**Fig 1:**
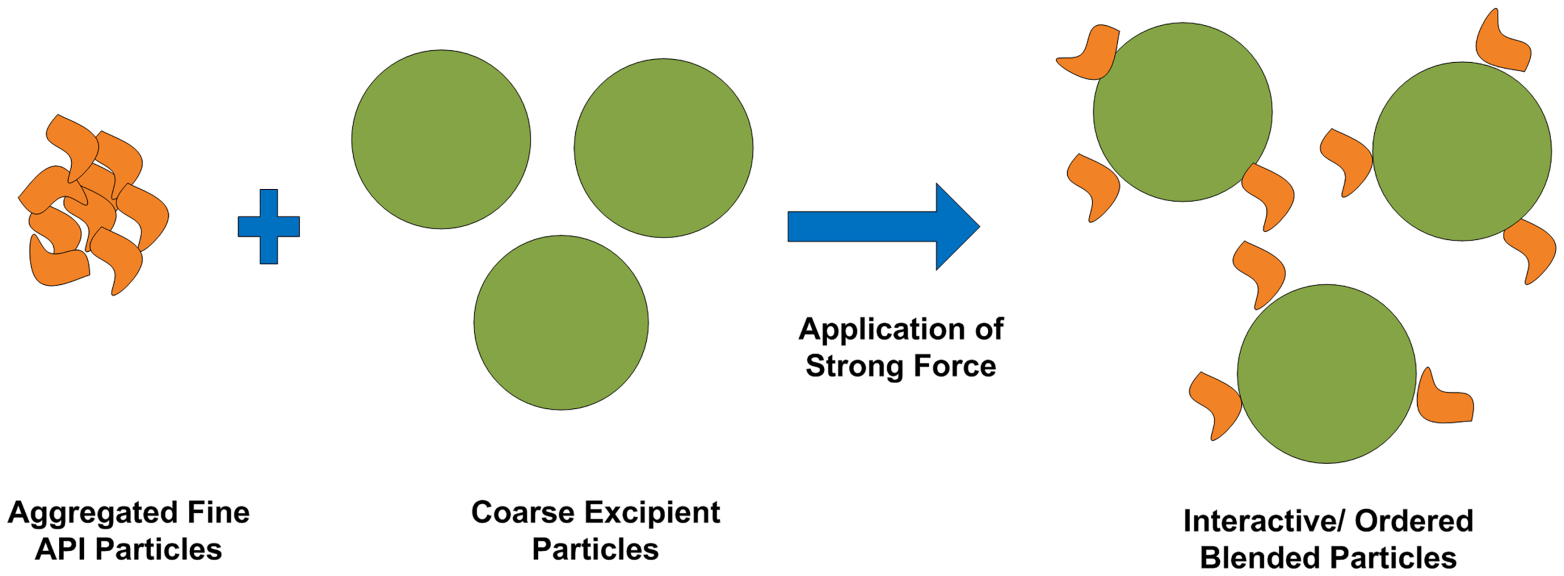
Schematic diagram showing the process of interactive/ordered blending. Fine aggregated API particles blended with coarse carrier/ excipient particles upon the application of strong mechanical force the fine API particles are deaggregted and get attracted to the surface of the excipient particles producing interactive/ ordered blended particles.

Various blending techniques to obtain homogenous blends for low API load have been reported. Apart from the multistep techniques like granulation and spray drying, geometric dilution is a commonly used technique when low load API formulation is developed. It implies gradual addition of equal portions of the diluent/ excipient to the API upon blending. The process increases the chances of equal distribution of the API particles within the blend. Ordered blending or interactive blending is another promising technique where fine API particles are adsorbed or attracted to the surface of coarse carrier/excipient particles (see Fig 1) that will facilitate homogenous blend and segregation prevention [2, 13, 14].

Obtaining optimal concentrations of excipients at the correct particle size and thereby enhancing flow is critical in improving manufacturability [15]. Particle size also has an impact on uniformity of content, with smaller particles allowing for more uniform blends to be achieved [16]. This produces a trade-off and the need for a balance to be found in order to produce powder blends with ideal characteristics for tableting whilst maintaining uniformity of content.

Microcrystalline cellulose (MCC), starch and pregelatinised starch are commonly used excipients in paediatric formulations. Their safety profile, low cost, high dilution potential and multifunctional role as fillers, disintegrants and binders enable their wide applications in solid dosage form formulations [17]. Further, the use of MCC pregelatinised starch is attaining popularity owing to their use in direct compression without the need for granulation [18].

The work in this study aims to investigate the impact of blending technique, excipient characteristics and dilution potential of three model excipients (starch, pregelatinised starch and microcrystalline cellulose) on developing a uniform blend comprising of small dose model API, ergocalciferol. To understand powder properties and their impact on product performance, excipients characteristics were tested using particle size analysis, angle of repose, scanning electron microscopy and interferometry. Both imaging techniques provided qualitative information that revealed surface characteristics of the used excipients.

## Materials and methods

### Materials

Starch and pregelatinised starch (starch 1500^®^) were obtained from Colorcon (Dartford Kent, UK). Ergocalciferol (Vitamin D2) was purchased from Discovery Fine Chemicals (Dorset, UK), whereas microcrystalline cellulose (MCC) Avicel PH-102 was donated by FMC BioPolymer Europe (Brussels, Belgium).

### Methods

#### Micronisation of Vitamin D2

Vitamin D2 was micronised by manual grinding using mortar and pestle for 30 minutes followed by sieving through different sieves for 12 minutes. 4 sieves (with 20cm diameter) were selected and weighed with the following mesh size range; 125 μm, 106 μm, 75 μm, 53 μm arranged as a nest according to size with coarsest on top using the vibratory sieve shaker Analysette- Spartan (Fritsch- GmbH) with deep amplitude (2.5mm). Then the fraction of particle size< 53 μm was manually passed through sieve with mesh size of 20 μm (particles with size ≤20μm were used for the study) in order to optimise distribution within powders and ensure better uniformity of content in the batches [19].

#### Sieving process

The original powders of the carriers (starch, pregelatinised starch and MCC) were sieved through different sieves. 8 sieves (with 20 cm diameter) were selected and weighed with the following mesh size range; 710 μm, 500 μm, 355 μm, 250 μm, 125 μm, 106 μm, 75 μm, 53 μm arranged as a nest according to size with coarsest on top. Sieving was carried out for for 12 minutes using the vibratory sieve shaker Analysette- Spartan (Fritsch- GmbH) at deep amplitude (2.5mm) in order to achieve separation of non-cohesive and cohesive parts of the powder. Approximately 40g of each powder was collected and labelled for use in the study. The particle size ranging between 125–180 μm was chosen as a non-cohesive fraction whereas particles with size with less than 53 μm were considered cohesive powders. Individual sieves were weighed to estimate the powder content. The process was repeated for additional 5 minutes to ensure weight difference did not exceed 5%.

#### Analytical technique

The amount of ergocalciferol dissolved in the solution samples was quantified using UV spectrophotometry (Jenway 6305 from Bibby Scientific Ltd. Staffordshire, UK) set at wavelength of 265 nm. The Method validation was investigated based on the International conference on Harmonization (ICH) guidelines for validation of analytical procedures [20]. Calibration curve was validated against specificity, linearity, accuracy, precision, limit of detection (LOD) and limit of quantification (LOQ).

#### Powder flowability (angle of repose method)

Powder flowability was evaluated using angle of repose method [21]. 5g of powders were poured through a funnel onto a flat surface. The funnel was positioned 10 cm from the horizontal surface, and the powders were allowed to flow freely until the formation of a symmetrical cone. Both the base (b) and height (h) of the formed cone were measured and recorded. Eq 1 was used to calculate the angle of repose (**θ**). Values were expressed as mean ± standard deviation (n = 3).

$$\theta =tan^{-1}\left(h/0.5b\right)$$ (1)

Results of the angle of repose correlate to flowability. Angle less than 30° indicates excellent flowability, between 31–35° is for good flowability, whereas angles above 45°is an indication of poor flowable powder [21].

#### Particle size analysis (laser diffractometer)

Powder particle size analysis was performed using laser diffractometer, Sympatec HELOS/ RODOS T4.1 (Clausthal-Zellerfeld, Germany). An R3 lens with a working range in between 0 and 178 μm was used for this study. The instrument permits the powder to circulate continuously through the system during the measurements through the sample dispersing system RODOS (dry disperser). Powder sample (0.5 g) was spread over the feeding tray of the VIBRI that transfers the sample into the dispenser (RODOS). Plots of particle size distribution are obtained covering the range from 0.5–175 μm. Parameters like the volume mean diameter (VMD), X_10_, X_50_ (median, 50% volume percentile) and X_90_ were obtained. The span of distribution was calculated using Eq 2. All the measurements were conducted in triplicate.

$$Span\;of\;distribution=\frac{\left(\times_{90}-\times_{10}\right)}{\times_{50}}$$ (2)

It should be noted that laser diffraction method produces high velocity for the powder which is aided by compressed air set at 3 bars that affects dispersion of the sample and hence, it is expected that the agglomerates originating from fine powder are dispersed into their primary particles and perfectly distributed [22].

#### Scanning electron microscopy (SEM)

SEM technique was used to study the morphological structure of particles. Samples were distributed by sprinkling on a double adhesive carbon tape placed over an aluminium stub. Then samples were coated twice with gold in a sputter coater Polaron SC500 (Polaron Equipment, Watford, UK) at 20 mA for three minutes and then examined by the SEM before imaging to enable sample conductivity. The sample imaging was performed on a field emission scanning electron microscope (Cambridge Stereo Scan (S90) Electron Microscope, Cambridge Instrument, Crawley, UK).

#### Surface topography using interferometer

Interferometric measurements of particles surface topography were performed using a MicroXAM2 interferometer (Omniscan, UK), operating using a white light source. Samples were imaged using a 50X objective lens. Scanning Probe Image Processor software (Image Metrology, Denmark) was used for the analysis of acquired images. Multiple images were tacked together to produce extended fields of average roughness in 3-D (Sa), root-mean-square roughness in 3-D (Sq), maximum height of the surface (Sy). The software enabled the calculation of adhesion energy and flowability parameters.

#### Blending techniques

Initial investigations focussed on developing formulations at four different weight ratios; 1:5, 1:10, 1:20, 1:50 w/w. Each of these would contain 10mg of API and 50, 100, 200 and 500mg of the carrier respectively, weighed using precise analytical balance then blended according to the different blending techniques.

#### Geometric blending technique

The first blending technique was based on geometric dilution. A stepwise geometric addition of excipient to the API was carried out to investigate the impact on content uniformity. The blending time was set either at one or five minutes. In this instance, each created batch was made of 600mg that was mixed in a 30ml screw cap tube. Three batches were prepared for each ratio and the process was repeated for each of the three carrier powders. All samples were prepared and 5mg of the blend was used for analysis of content uniformity using UV spectroscopy.

#### Manual ordered blending technique

600mg batch size at 1:50 ratio was carried forward for evaluating the impact of manual blending on content uniformity. This consisted of addition of the total quantity of the excipient in one step into the sample tube after the required amount of API had been added. 5mg was taken from the blended powder over different time points (0, 2, 4, 8, 16, 32 minutes) for content analysis. The process was repeated for each of the three carrier powders.

#### Ordered blending using dry powder coater

**Fig 2:**
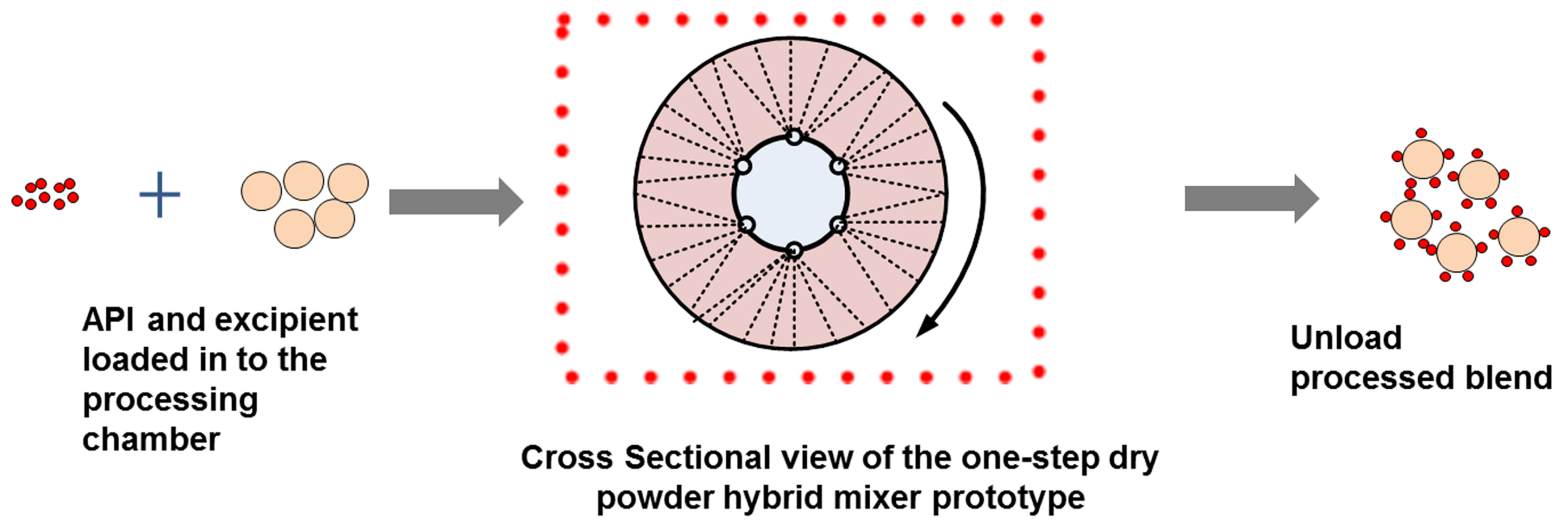
Schematic showing the process of dry powder hybrid mixing. Fine guest material (API) and coarse carrier (Excipient) are added to the high G-force processing vessel coupled with air blade (nitrogen gas) under highly controllable conditions to produce final interactive blend.

The ‘dry powder hybrid mixer prototype’ was assembled by the research group at Aston University. The machine is designed to supply sufficient mechanical force essential to break the agglomerates that are created by cohesive powder and promote ordered and structured blending of powder blends. The machine comprises of a high speed rotating motor which has a speed ranging between 300–2000 rpm which is linked to a rotating container by means of a smooth inner surface in which the powder (API as well as carrier/excipient) is enclosed. To assist collision external air supply is provided via nitrogen gas which is linked to the blending container providing an air blade (see Fig 2).

A final investigation was performed using ordered addition of non-sieved carrier which comprised of six batches made up to 3 g, containing 1% w/w API and 0.5% API. The rational for the selection of lower concentration (0.5% and 1%) is to meet the requirements for low dose of paediatric formulations. Blending was done by the same device at 300rpm for a total 32 minutes. 5mg samples were taken from the blended powder over a range of time points (0, 2, 4, 8, 16 and 32 minutes) to assess blend homogeneity.

#### Drug content uniformity using UV analysis

5 mg of the blended powders of ergocalciferol and carriers at 1:5, 1:10, 1:20, and 1:50 were dissolved in 50, 25, 10, 5ml respectively of ethanol. The solution was filtered through a 0.45 μm nylon filter (CHROMACOL LTD, Herts., UK). Ergocalciferol content uniformity was assayed using spectrophotometer using UV analysis. Spectrophotometer at wavelength 265 nm was used [21, 23]. Values are expressed as mean ± standard deviation (n = 9).

#### Statistical analysis

Statistical analysis studies were done using Graph Pad Prism software (Version 3.01, CA, USA). As applicable, t-test, one way analysis of variance (ANOVA) and pair-wise multiple comparisons method (Tukey’s test) were used to compare data groups by using mean values and standard deviation (SD). The significant difference was determined using the probability value of 95% (P < 0.05).

## Results and discussion

### Analytical technique

Calibration curve was prepared from dilutions of stock solution of ergocalciferol (10 μg/ml), using ethanol as solvent. The absorbance of the dilutions of stock solutions was determined by UV spectrophotometer, at wavelength of 265 nm. Six points calibration curve was obtained in a concentration range from 0–3.6 μg/ml for ergocalciferol. The response of the drug was linear in the investigation concentration range and the linear regression equation was y = 0.0443x with correlation coefficient R^2^ = 0.999. Summary of the validation parameters is presented in Table 1

**Table 1:** Summary of the validation process parameters for ergocalciferol using UV spectrophtometre at 265nm.

Validation Parameter	Intraday % Recovery [mean ±SD, (RSD)]			Inter-day % Recovery [mean ±SD, (RSD)]		
	2 μg/ml	5 μg/ml	10 μg/ml	2 μg/ml	5 μg/ml	10 μg/ml
**Accuracy** **(n = 3)**	101.1±2.01	98.67±1.01	99.18±3.802	-		-
**Precision** **(n = 6)**	101.2±0.81 (0.80)	-	100.84±0.78 (0.77)	98.03±1.74 (1.77)	-	96.77±2.11 (2.18)
**Validation Parameters**						
**Slope**		0.0443		**Residual Standard Deviation standard deviation**	0.0021	
**Standard regression equation**		y = 0.0443X		**LOD (μg/ml)**	0.16	
**Regression Coefficient (R2)**		0.999		**LOQ (μg/ml)**	0.48	

### Powder characterisation

In-depth analysis of powder properties enables understanding or even predicting their performance upon blending with API. The primary aim of this study was to investigate the impact of particle characteristics on blending small quantities of candidate API. The classification of the powders into cohesive and non-cohesive was done to identify the impact of particle size during the process of blending on dose uniformity. The study commenced with the evaluation of particle size measurements and was followed up with powder flow, scanning electron microscopy and interferometry studies.

Understanding powder flow is a critical attribute during pharmaceutical manufacturing processes like, blending, packaging, transportation, tabletting and capsule filling. Therefore, it becomes vital to determine the flow properties of powders for processability [24].

There are two classes of material properties that need to be considered in blending powders. The first is the cohesive materials that tend to aggregate leading to flowability issues while the second class is the non-cohesive free flowing materials that could demonstrate excellent flowability but can lead to occasional segregation [25–28].

Particle size analysis results as depicted in Table 2 showed that the average particle size of starch is around 11.9±0.51 μm which is expected from a cohesive fine powder. Optimising drying conditions to produce gelatinisation of starch can cause the particles to adhere together to form aggregates [29] and hence the average particle size of pregelatinised starch exhibited a remarkable increase of VMD to 79.75±1.49 μm with a wider span of distribution (1.38±2.48). With regard to MCC, using the laser diffraction particle size results produced an incomplete distribution curve as the powder contains particles with size exceeding 175 μm. Similar results were obtained for ergocalciferol (VMD: 73.66±5.18 μm). According to Zhang et al, who analysed whether drug particle size or else mixing is accountable for poor content uniformity, it was noted that decreasing particle size enhances content uniformity provided drug aggregation is controlled [19]. Therefore, Ergocalciferol was micronised to enhance content uniformity in this study. The results showed a reduction of MVD to 13.7 ±0.42 to enable blend homogeneity.

**Table 2:** Flow properties of starch, pregelatinised starch (P.starch), MCC, ergocalciferol (Erg.) and micronised ergocalciferol (Mic Erg.) showing the particle size analysis parameters (X10, X50, X90, Span and volume mean diameter (VMD)), angle of repose (°) and the corresponding flow property.

Product	X10	X50	X90	Span	VMD (μm)	θ (°)	Flow property*
Starch	7.72±0.62	11.75±0.43	16.65±0.33	0.761±0.05	11.9±0.51	42.46±2.5	Passable
P. Starch	27.72±2.46	77.24±2.52	134.27±3.39	1.38±2.48	79.75±1.49	34.90±2.2	Good
MCC	18.65±0.29	62.75±2.07	141.26±0.66	1.96±0.05	73.05±0.89	28.0±1.44	Excellent
Erg.	9.22±1.31	67.52±13.15	142.10±3.76	2.07±0.33	73.66±5.18	34.98±1.98	Good
Mic Erg.	1.10±0.09	8.46±0.46	26.89±0.83	3.06±0.23	13.70±0.42	41.35±1.99	Passable

*Excellent flow when angle of repose (θ): 25–30°; Good: 31–35°; Fair: 36–40°; Passable 41–45°; Poor: 46–55° [19]

**Fig 3:**
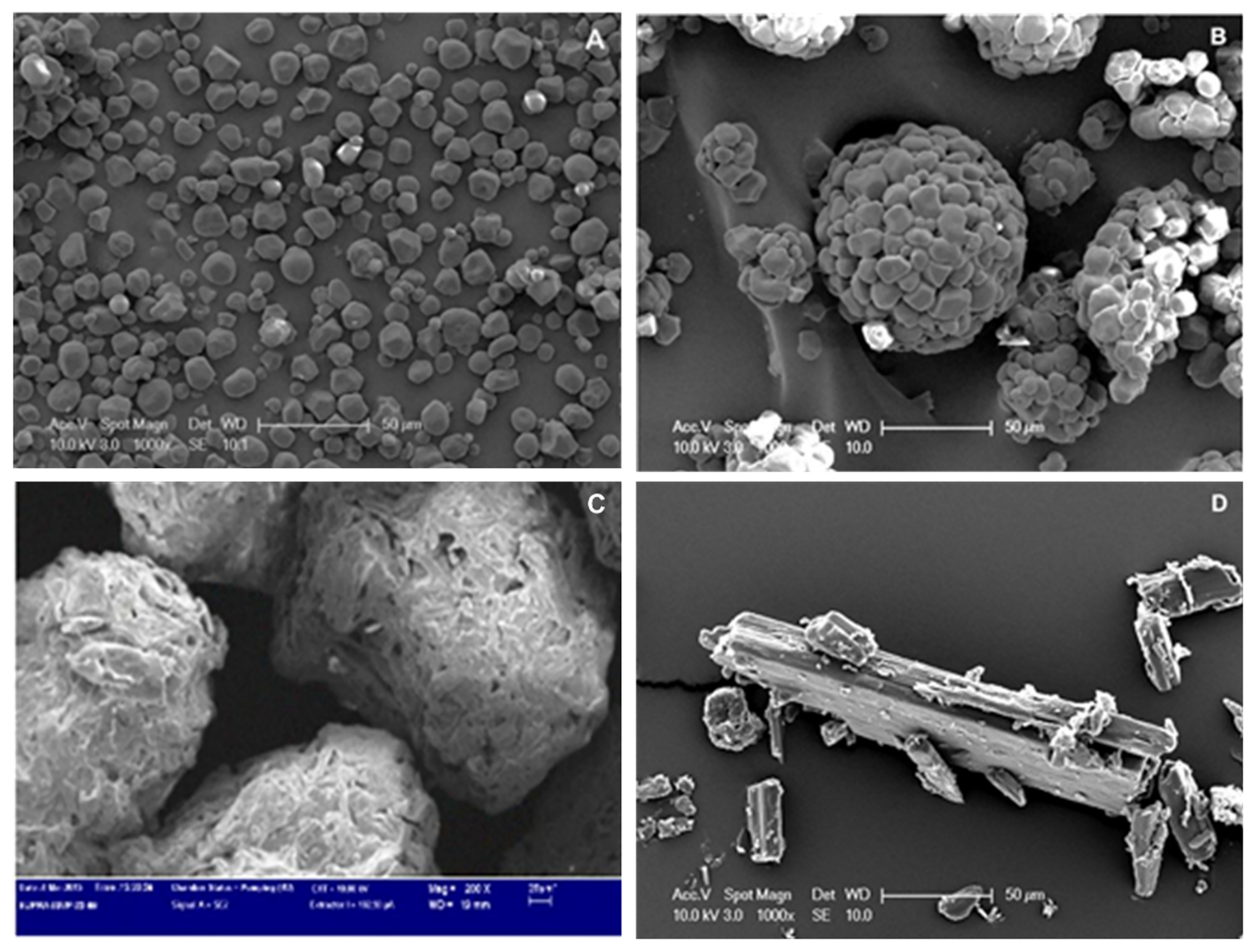
Scanning electron microscopy micrographs at 1000 times magnification of (a) starch (b) pregelatinised starch (c) MCC and (d) ergocalciferol.

The results of flowability based on angle of repose showed that apart from starch and micronised ergocalciferol, flowability was good to excellent for the non-sieved materials. Owing to its small particle size, starch demonstrated low flowability profile. This was further evident from the SEM images that showed individual particles as well as cluster of granules. Starch particles were smaller in size and had typical angular, spherical or rounded shape with smooth surface (Fig 3A). Pregelatinised starch SEM images (Fig 3B) showed larger clusters of particles which explains enhanced flowability when compared to starch. On the other hand, excellent flowability of MCC could be attributed to the granular particle shape and particle size that is larger than the estimated using laser diffraction as evident from the SEM (Fig 3C). The general shape for ergocalciferol can be described as longitudinal and irregular with roughness surface (Fig 3D).

As the aim of the work was to further investigate powder behaviour upon blending and the performance of blend, the three excipients (pregelatinised starch, starch and MCC) were sieved and the cohesive part of the powder (particle size less than 53 μm) as well as non-cohesive part where the size of the particles was between 125–180 μm were collected and further investigated except for starch only non-sieved and cohesive portions were used as the particle size of starch was low. Flow properties of non-cohesive pregelatinised starch were found to be excellent as depicted in Table 3. Similarly as expected, cohesive fractions of both starch and pregelatinised starch showed poor flowability due to their smaller particle size. The non-sieved and non-cohesive MCC fell into the excellent flow properties category whereas cohesive MCC had fair flow. The results obtained were statistically significant (p<0.05, one way ANOVA) as larger particles have better flow properties whereas smaller particles due to increased Van der Waals interactions between particles exhibited poor flowability therefore it follows that the cohesive powder would have a higher angle of repose compared to the other two types of MCC. The non-cohesive MCC and non-sieved had similar flow properties due to particles around 200μm generally having excellent flow properties [30].

**Table 3:** Flow properties of cohesive starch, cohesive and non-cohesive pregelatinised starch and cohesive and non-cohesive MCC showing the particle size range (obtained upon sieving), angle of repose and the corresponding flow property.

Carrier (particle size)	Particle size range (μm)	Angle of repose (°)	Flow property
Cohesive starch	<53	49.30 ± 0.59	Poor
Non-cohesive pregelatinised starch	125–180	30.14 ± 2.3	Excellent
Cohesive pregelatinised starch	<53	48.44 ± 4.2	Poor
Non-cohesive MCC	125–180	25.0 ± 0.86	Excellent
Cohesive MCC	< 53	39.46 ±1.29	Fair

An attempt to further understand the excipients characteristics was studied using interferometry. The technique provides an in-depth analysis of the surface properties and topography parameters as summarised in Table 4. The first parameter is the average surface roughness (Sa) for each sample and the results showed high surface roughness for MCC followed by pregelatinised starch and starch. Sieving to obtain the fine fraction of material resulted in a significant increase in surface roughness for pregelatinised starch (t-test, p<0.05) whereas starch showed slight reduction in roughness. The increase in roughness upon sieving could be attributed to the increase in surface area with lower particle size as the average size of pregelatinised starch dropped from 40 to 25μm. The change in particle size upon sieving for starch was minimal and the particles were <10 μm.

**Table 4:** Surface topography parameters and flow properties of the main excipients showing average roughness (Sa), mean square value of average roughness (Sq), maximum roughness height (Sy) particle radius (R), adhesion energy (AE), angle of repose (*θ*) and flow property (FP) (mean ± SD, n = 5).

Material	Sa (nm)	Sq (nm)	Sy (nm)	R (μm)	AE (aJ)	(*θ*)	FP
Cohesive MCC	875±60	1230±115	9604±105	26.5	28.5	39.46±1.29	Fair
Non-sieved starch	126±38	168±60	1700±103	6.0	114.5	42.46±2.5	Passable
Cohesive starch	81±34	109±46	703±156	3.5	108.5	49.3±0.59	Poor
Non-sieved pregelatinised starch	174±53	233±80	2934±160	40.0	251.5	34.9±2.2	Good
Cohesive pregelatinised starch	264±109	390±212	5216±173	25.0	153.6	48.44±4.2	Poor

In this study the high surface roughness of MCC was anticipated to enhance content uniformity compared to starch and pregelatinised starch. It was expected that fine API particles will be lodged into and between the rough surface apertures of MCC and hence enhance uniformity. It was reported that the higher the degree of roughness, the better content uniformity of the blend [31]. Therefore, based on particle size and surface roughness, MCC showed the best uniformity followed by pregelatinised starch and starch. Further, starch was expected to produce poor content uniformity owing to the small particle size (3.5–6 μm) and cohesive nature of starch that showed high cohesive energy (~110aJ). The second parameter from Table 4 is the *Sq*. It is the root mean square value of the surface roughness within the sample area. It is considered as a more statistically significant parameter than *Sa* as Sq has the effect of giving extra weight to the numerically higher values of surface height [31, 32]. Similar to the results obtained from Sa values, MCC particles showed higher Sq values than that of pregelatinised starch or starch.

The third parameter Sy is the maximum height of surface. It is the sum of the height of the largest peak height value and the largest valley depth within the sample. Examining both the *Sq* value and the *Sy* value, an indication of whether the apparent roughness is due to isolated features or the overall surface roughness can be derived [31, 33].

**Fig 4:**
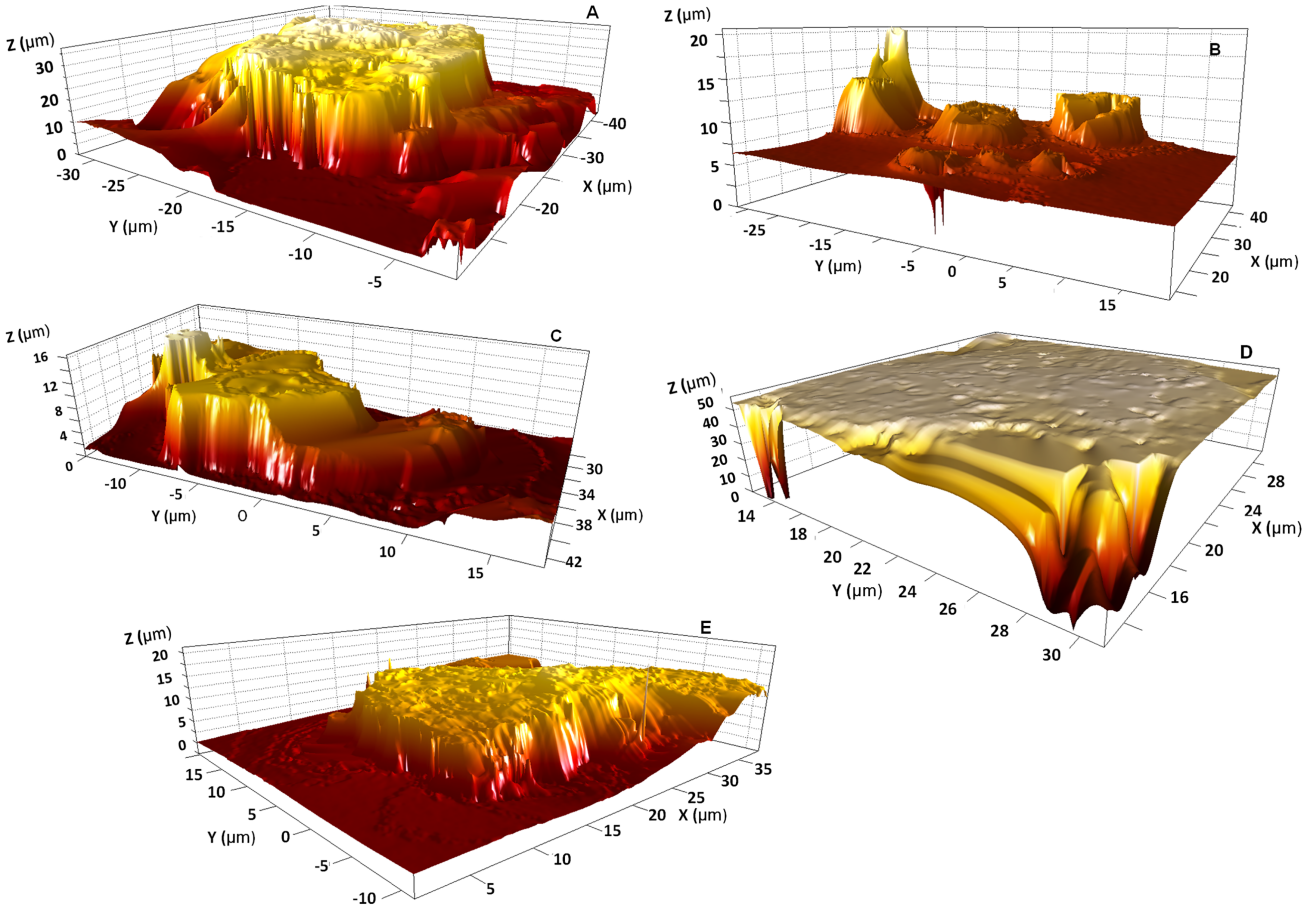
Interferometer topographical images of (A) cohesive MCC, (B) non-sieved starch, (C) cohesive starch, (D) non-sieved pregelatinised starch and (E) cohesive pregelatinised starch.

Results for all the samples showed that the Sy value were different compared to that of Sq value which indicated that the topography is irregular as evident from the 3D images in Fig 4. On the other hand, the change in Sy value for MCC particles was of a greater magnitude and provides a strong evidence of the degree of roughness compared to the other excipients. Surface energy parameters as depicted in Table 4 showed the change in adhesion energy for different materials. The lowest was noted for cohesive MCC with particle size of around 26 μm and adhesion energy of 28.5 aJ. Such low energy for MCC possibly explains fair flowability despite the small particle size (39.46±1.29). It is reported that particles of less than 50 μm are highly cohesive [2]. This also could be further correlated to surface roughness (Sa) where high roughness values will result in less surface contact between particles and hence less cohesiveness with better flowability to produce homogeneous blend. The cohesive energy for pregelatinised starch and starch were related to the change in surface roughness of material and hence could justify that higher roughness is related to lower cohesive energy however, that could not clearly relate to flowability as the particle size of pregelatinised starch and starch were very low.

### Investigation of various blending techniques and their impact on drug content uniformity

Ergocalciferol is a suitable model API as the maximum dose is generally around 750–800 micrograms per tablet. The different ratios of drug to carrier were selected to simulate dilution of drug during tablet development when blended with different excipients. Starch, pregelatinised starch and MCC are routinely used in tablet manufacturing and represent a pragmatic choice to investigate the above hypothesis.

Having investigated material properties for the model drug as well as the different particle fractions for the three carriers, the next objective was to study the impact of different blending techniques primarily on the content uniformity of the drug. Firstly, geometric blending was employed to study the impact of powder content and duration of blending on blend homogeneity. Secondly, ordered blending using two different approaches including manual blending and high speed blending were investigated.

#### Geometric blending technique

Geometric blending is a standard technique to blend small amounts of API and according to British Pharmacopeia (BP) [34], drug content for ergocalciferol should range between 90 to 120% of the label claim. Four formulations were prepared for geometric blends (drug: carrier ratios: 1:2, 1:10, 1:20 and 1:50). All blends were prepared with a total batch size of 600mg.

#### One minute geometric blending

**Fig 5:**
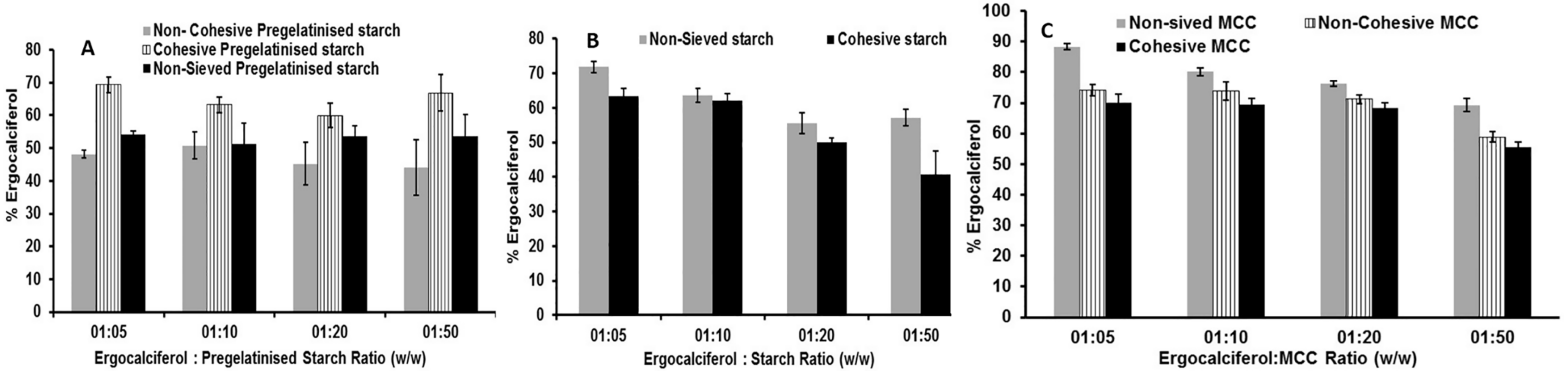
Influence of carrier: Drug ratio and carrier particle size on content uniformity of ergocalciferol. Blends are made of ergocalciferol and (A) pregelatinised starch (B) Starch & (C) MCC (non-sieved, cohesive and non-cohesive) blended at various ratios (1:5, 1:10, 1:20 and 1:50) using geometric blending for 1minute (mean± SD, n = 9).

The first set of studies focussed on the impact of particle size characteristics of pregelatinised starch on content uniformity. The results presented in Fig 5A showed that blending for one minute using different particle size fractions of pregelatinised starch in various ratios resulted in poor drug recovery. Although the deviation increased with the increase in dilution, all batches failed the content uniformity test. Similar trends were observed for non-sieved as well as cohesive blends. Despite the failure to meet the required pharmacopeia standards for drug recovery, the results showed some interesting trends. It would be expected that cohesive powders would potentially result in non-uniformity due to particle aggregation whereas non-sieved powders would generate a more uniform drug blend. From the current study, it can be clearly seen that the recovery was the least from non-cohesive blends followed by non-sieved and cohesive powders. Although the cohesive blends provide greater surface area for particle interaction, the short blending duration (1 minute in this case) ensures that particle aggregation is controlled thereby resulting in a slightly better distribution and recovery of the drug. The better content uniformity of cohesive pregelatinised starch could also be attributed to the higher surface roughness and lower adhesion energy (Table 4). Lower adhesion energy is attributed to the presence of asperities that reduce the contact surface area between particles. On the other hand, despite the good flow properties of the other two blends, uneven particle distribution possibly resulted in segregation and low drug recovery. One-way ANOVA test for the data set demonstrated that p value was <0.05.

Studies investigating dilution and blending using starch showed similar results as shown in Fig 5B. The two grades of starch (with different particle sizes) in the various ratios tested resulted in low drug recovery. All the batches that were tested failed the regulatory requirement. The results showed that the percentage drug recovery decreased with an increase in dilution of the drug with the carrier. The cohesive blend of starch resulted in lower drug uniformity with the increase in dilution compared to non-sieved this could be attributed to the high cohesive nature of the starch.

There was a significant difference between the different ratios for MCC (p<0.05, one way-ANOVA) as illustrated in Fig 5C. Further, the non-sieved grade was superior to the other grades in terms of content uniformity. Overall, geometric blending for one minute for all carriers including pregelatinised starch, starch as well as MCC did not meet the required uniformity range.

#### Effect of increasing blending time on drug content uniformity

Based on the results achieved from the earlier experiment, the time of blending was elevated up to five minutes after each addition of excipient. The rationale was to determine if longer blending time would promote uniform distribution and aid diffusion of the API particles within the carrier particles. The longer the time for blending, greater is the time of contact between all the particles and this may induce better homogeneity due to increased chances of collision [16].

**Fig 6:**
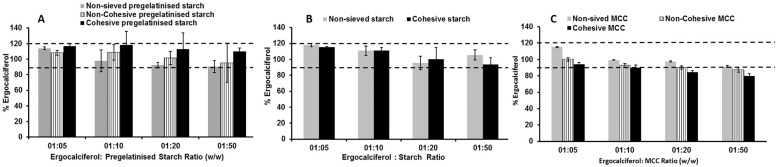
Influence of carrier: Drug ratio and carrier particle size on content uniformity of ergocalciferol. Blends are made of ergocalciferol and (A) pregelatinised starch, (B) Starch & (C) MCC (non-sieved, cohesive and non-cohesive) blended at various ratios (1:5, 1:10, 1:20 and 1:50) using geometric blending for 5 minute (mean± SD, n = 9).

The results obtained in Fig 6A showed that drug recovery was complete and all the tested ratios showed that prolonged blending time resulted in complete drug recovery. The results showed no significant difference (one-way ANOVA, p>0.05) between the different particle size portions of pregelatinised starch. Similar trends were obtained when pregelatinised starch was replaced with starch (Fig 6B).. It can be hypothesised that the increase in blending time promotes better distribution of the drug irrespective of the particle characteristics and the type of the carrier. The process of powder blending is influenced by different forces including diffusional, shear and convective forces. In the method employed for geometric blending, it can assumed that the impact of shear forces would be minimal as the powder particles are not subjected to higher magnitude forces as that would be obtained in fluidised bed or high speed blenders. It is likely that the resultant uniform blend of the powder blend for both the carriers with different particle size fractions could be due to the combination of diffusion and convective blending. In the case of non-sieved and free flowing powder blends, convective blending ensures the transport of powder bed from one location to the other. This allows for the movement of large particles from one area of the blending tube to the other generating a random blend. The results from the investigation above where the duration of blending was one minute possibly initiated the transfer of the bulk powder without the generation of a consistent random blend. Following the increase of the duration of blending, it is likely that the movement of powder bed through convection increases the distribution of the particles of the drug between the carrier particles resulting in the generation of a random blend. The longer duration of blending also ensures that diffusional blending promotes movement of particles at the micro-level thereby enhancing content uniformity. The longer duration of powder blending results in prevention of de-blending which can take place before an ultimate random state is obtained. Cohesive blends on the other hand presented a different set of challenges. Although the increase in blending time resulted in a uniform blend for both the carriers containing cohesive powders, the outcomes can be explained based on the processes that occur during dry cohesive blending.

Previous research has shown that cohesive blends segregate due to multiple factors including particle shape, size and the intensity of cohesion [35]. The process of blending for cohesive powders relies on not only the properties of the cohesive particles but also on the adhesive interactions in a binary mixture. Simulation studies for dry blending of cohesive mixtures by [36] confirmed that the extent and intensity of the cohesive forces between similar particle sizes controls the degree of segregation which ultimately impacts on the homogeneity of the powder blend. The studies showed that the presence of low intensity cohesive forces promotes blending possibly due to the larger surface area available for the particles within the binary mixture to achieve a random state of distribution. In our study, it can be concluded that the intensity of the cohesive forces between the particles for both the carriers is relatively weak thereby ensuring that aggregation between similar particles is reduced and therefore promotes a more uniform distribution of the particles of the drug. Interestingly, the non-sieved MCC formulation displayed ideal uniformity whereas the non-cohesive and cohesive formulations had slightly lower uniformity as shown in Fig 6C. The difference between these formulations was significant (p<0.05), indicating that at this decreased concentration, increasing blending time still showed benefit for obtaining uniformity with the largest particle size. The superiority of non-sieved MCC also can be attributed to lower segregation tendency compared to non-cohesive as well as the less adhesive energy when compared to cohesive MCC. Therefore, it became evident that geometric addition at 5 minute intervals serves to improve uniformity for highly dilute blends (1:50) compared to 1 minute blending [37].

#### Ordered blending technique

Following investigations of geometric blending, ordered blending using bulk powder (as opposed to incremental addition in geometric dilution) was investigated to study the impact on drug content uniformity. Ordered bulk blending represents a more convenient method from an industrial perspective due to the availability of a wide range of equipment, being one step process and relatively shorter processing time.

#### Ordered blending using manual blending

The objective of this study was to determine the time dependent effect on content uniformity during bulk blending using a manual blending technique. The 1:50 ratio was chosen for this technique as it represents the maximum dilution potential for the drug that was used in our previous studies. This technique was carried out to find the relationship between percentage drug recoveries with respect to time. Blending was performed until a constant relative standard deviation for drug content was obtained. Similar to the above investigations, the different particle size ranges for the three carriers were investigated.

**Fig 7:**
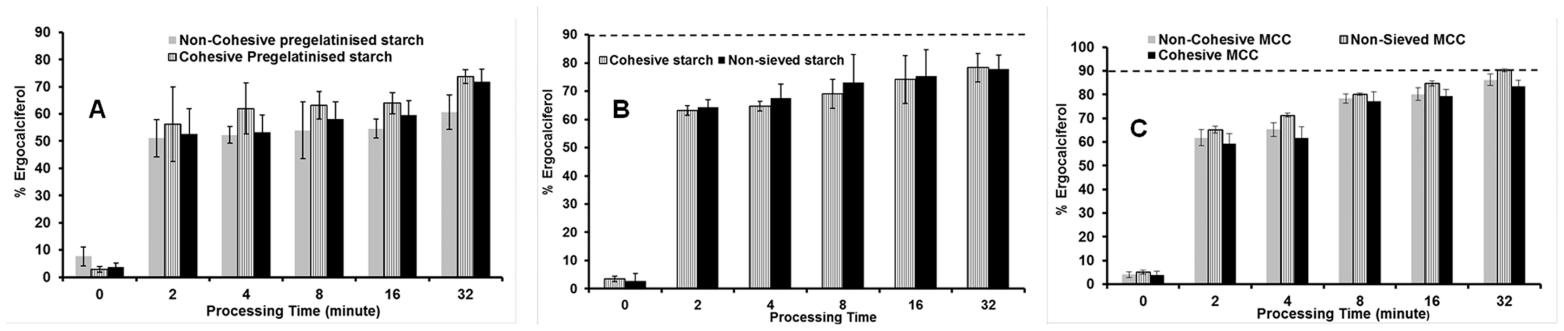
Influence of processing time and carrier particle size on content uniformity of ergocalciferol. Blends are made of ergocalciferol and (A) pregelatinised starch, (B) Starch & (C) MCC (non-sieved, cohesive and non-cohesive) blended at drug: carrier ratio of 1:50 using vigorous hand blending technique (mean± SD, n = 9).

The results in Fig 7A demonstrate that content uniformity increased with blending time with all forms of pregelatinised starch and the highest percentage drug content uniformity was achieved after 32 minutes for all three forms of pregelatinised starch. Cohesive pregelatinised starch showed the highest percentage of drug recovery at various time points when compared to the non-cohesive blends. For example, after half an hour of blending, cohesive powder of pregelatinised starch showed highest content uniformity of about 75% while non cohesive was lowest around 55% (one way ANOVA, p<0.05). Interestingly these results are similar to that obtained using geometric blending where the cohesive blends of pregelatinised starch outperformed the non-cohesive blends despite all the three grades not achieving the pharmacopeia standards.

**Fig 8:**
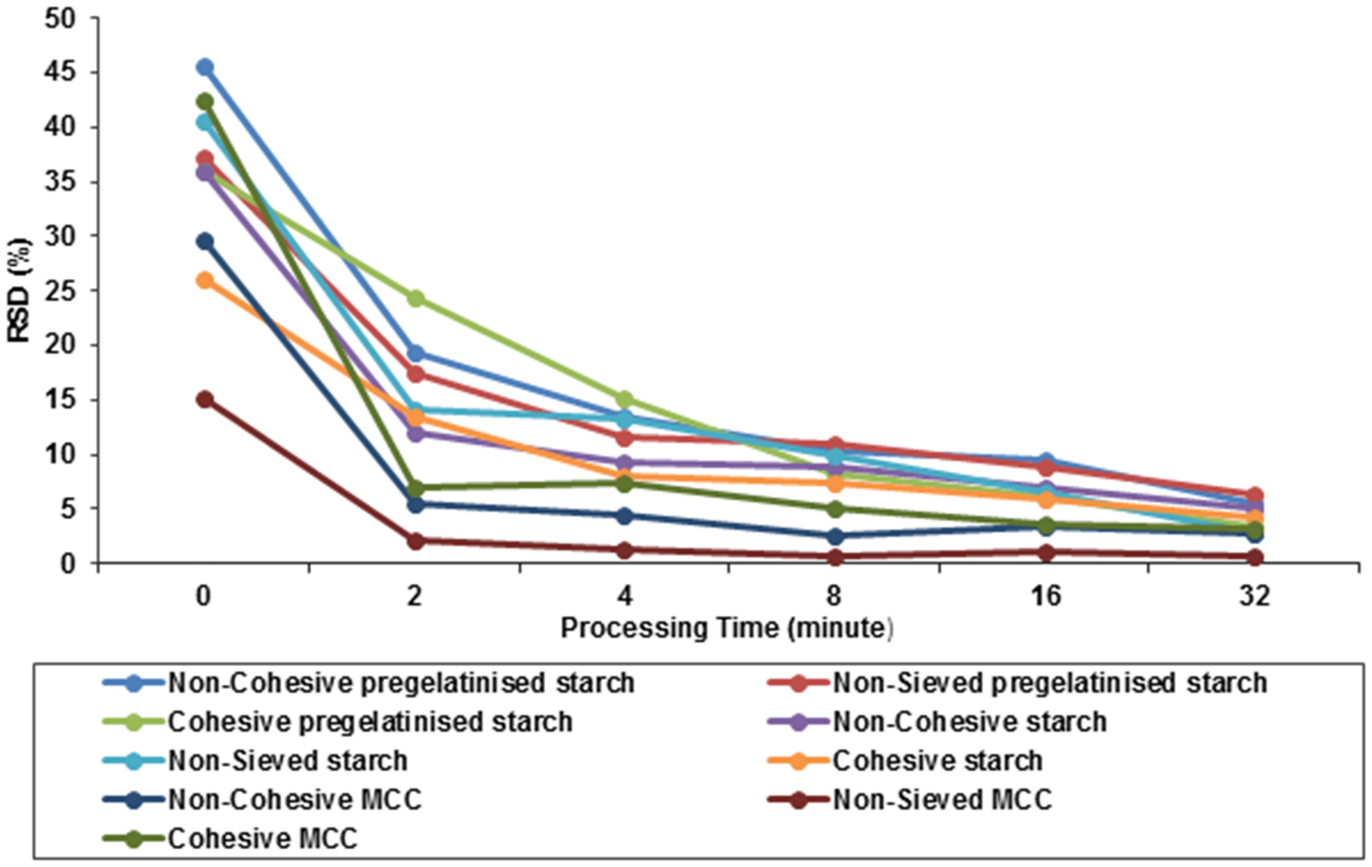
Influence of processing time, carrier type and carrier particle size on blend homogeneity as expressed in RSD. Blends are made of ergocalciferol and carrier blended at drug: carrier ratio of 1:50 using vigorous hand blending technique (n = 3).

In case of starch with ergocalciferol as shown in Fig 7.Bit was observed that the increase in percentage of drug recovery was exhibited by non-sieved starch (one way ANOVA, p>0.05). The non-sieved MCC reached the lowest acceptable level of uniformity at 32 minutes of blending as shown in Fig 7C. However, smaller particles allowed for faster achievement of uniformity within a powder blend [38]. It is evident from the graph that the relative standard deviation for all the samples as shown in Fig 8 followed a similar pattern. The results showed that the standard deviation began to plateau just after five minutes and there was no difference until the conclusion of the study.

Using this type of blending, all batches for the three carriers (except non-sieved MCC) did not achieve the requirements of BP for drug content uniformity of ergocalciferol. Fig 8 shows relative standard deviation (RSD) for ergocalciferol based on 1:50 pregelatinised starch, starch and MCC at different time points. It was observed that RSD for ergocalciferol was the lowest for non-sieved MCC which showed an initial value of 15% at 0 minute which decreased to approximately 1% after 32 minutes. Hence, non-sieved MCC had the lowest standard deviation compared to all carrier formulations. The graph demonstrates that the drug distribution for the different types of blends requires longer duration to obtain more homogenous blend. The higher deviation at the start indicates that the drug is concentrated in various pockets comprising of “drug-concentrated” areas which need to be relocated within the bulk of the diluent. Continuous blending ensures that convective blending predominates and the drug has the opportunity to distribute itself between the carrier particles. The standard deviation begins to plateau after about five minutes which possibly suggests that the drug rich domains have been redistributed randomly within the bulk of the carrier. It is possible that after the first five minutes, diffusional blending predominates and therefore micro blending of the drug between the particles is the key factor. Despite the expected cohesive forces between the smaller particles fractions which can promote aggregation, it is possible that weak/lower intensity forces operate which ensure that diffusional micro blending predominates over the cohesive interactions.

#### Ordered blending using dry powder hybrid mixing device

The last method of blending included investigation of a dry powder hybrid mixing device as a technique to generate homogenous powder blends. Hersey (1975) and Ishizaka (1989) [39, 40] described “ordered blending” as cohesive powder blending process where fine particles are dispersed and get attached to the surface of coarse particles to generate an ˝interactive mixture˝.

The blending device used in this study was built at Aston University. In the current study the speed of the device was fixed at 300rpm with no air injection. The speed was chosen after optimisation studies which showed that higher speeds lead to the formation of dry coated particles whereas lower speed promotes homogenous blending and formation of interactive/ordered blend. In interactive blending using dry powder hybrid mixing device, the blending process depends on two forces. The adhesion forces of the API to the carrier particles and the cohesion forces between the drug particles. Proper blending will be achieved and no agglomerates will be left only if the adhesion force between materials is greater than the cohesive forces between similar particles [41]. Based on the results obtained from ordered blending as described above, the dry powder hybrid mixing device at speed 300 rpm was investigated in order to promote uniform distribution of the drug between the carrier particles.

**Fig 9:**
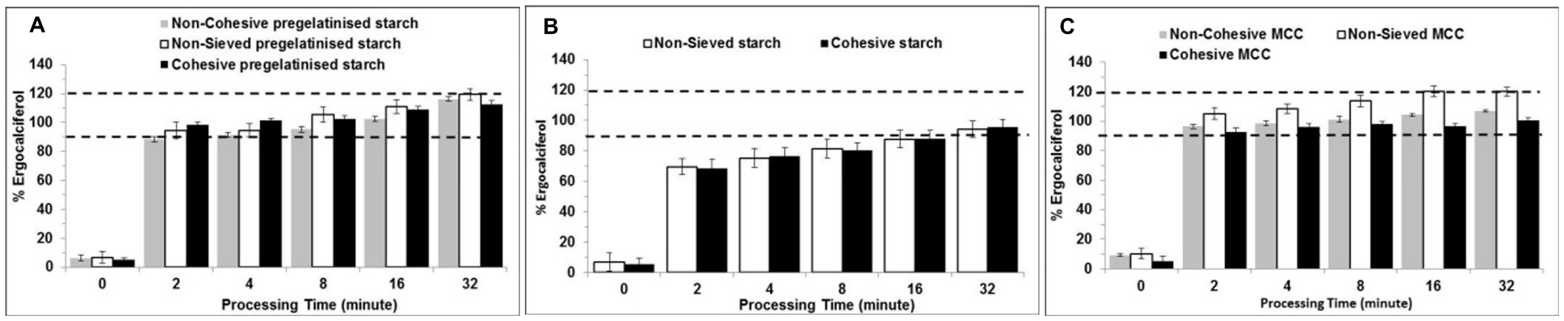
Influence of processing time and carrier particle size on content uniformity of ergocalciferol. Blends are made of ergocalciferol and (A) pregelatinised starch (non-sieved, cohesive and non-cohesive) (B) starch (non-sieved and cohesive) and (C) MCC (non-sieved, cohesive and non-cohesive) blended at drug: carrier ratio of 1:50 using interactive blending technique (mean± SD, n = 9).

The results obtained in Fig 9 showed that drug recovery was achieved after 4, 32 and 2 minutes of blending for all blends of pregelatinised starch, starch and MCC respectively. The reason behind the enhancement of drug uniformity is a result of sufficient blending process that included different forces such as convective, shear and diffusional forces. Two-way analysis of variance (ANOVA) test and Tukey’s test demonstrated a statistically significant difference between batches and between each batch with different times (p<0.05). It can be hypothesised that the impact of shear forces was achieved after 16 minutes of blending, so that the deviation sharply decreased with all forms of carriers at 32 minutes of blending.

**Fig 10:**
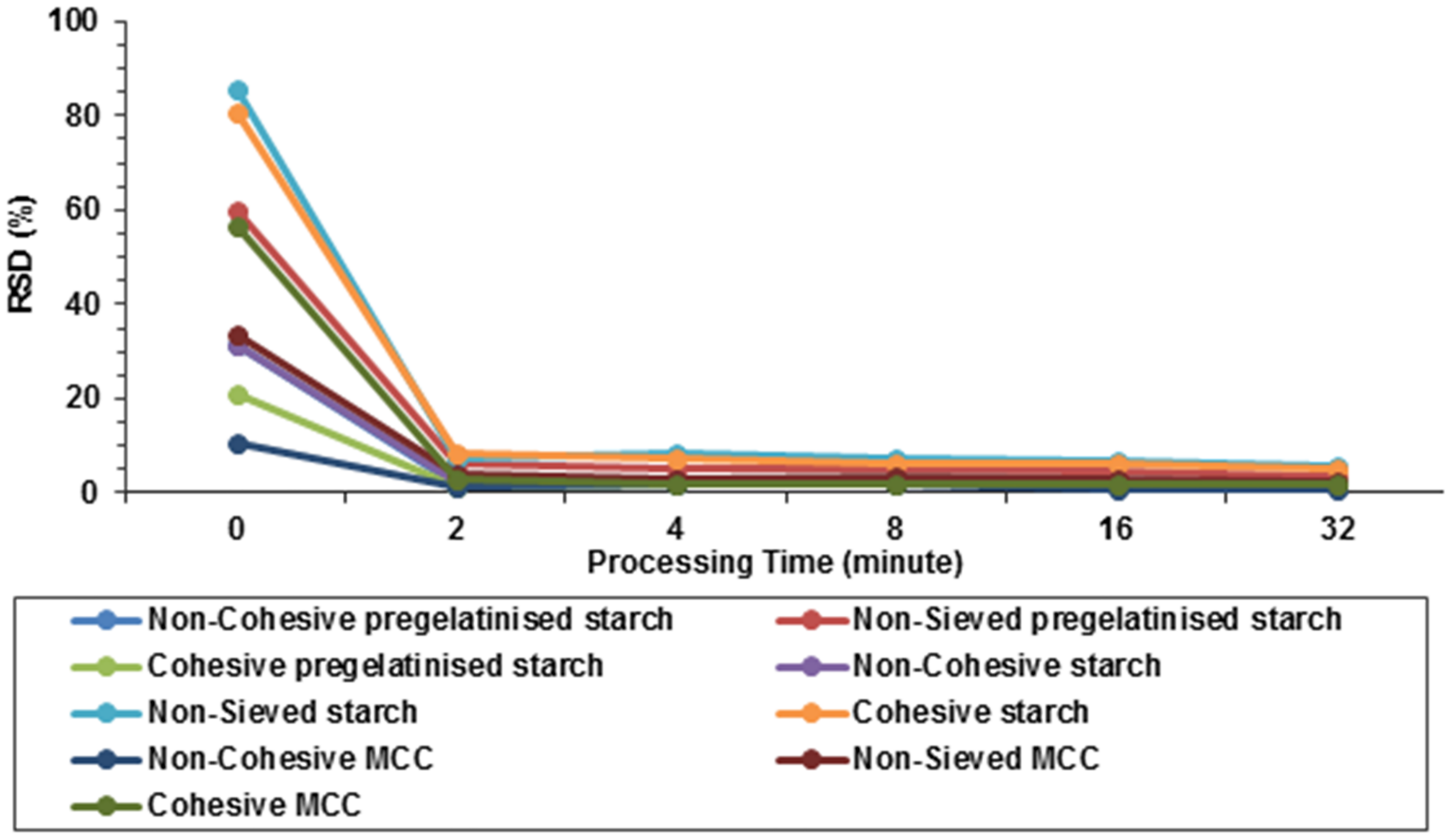
Influence of processing time, carrier type and carrier particle size on blend homogeneity as expressed in RSD. Blends are made of ergocalciferol and carrier blended at drug: carrier ratio of 1:50 using interactive blending technique (n = 3).

From Fig 10, it was seen that the RSD for ergocalciferol was the highest for non- sieved and cohesive starch which showed an initial RSD of 85% and 80% at 0 minute and decreased to approximately 5% in 32 minutes.

#### Blending of 0.5% and 1% of API: Non-sieved carrier using dry powder hybrid mixing device

**Fig 11:**
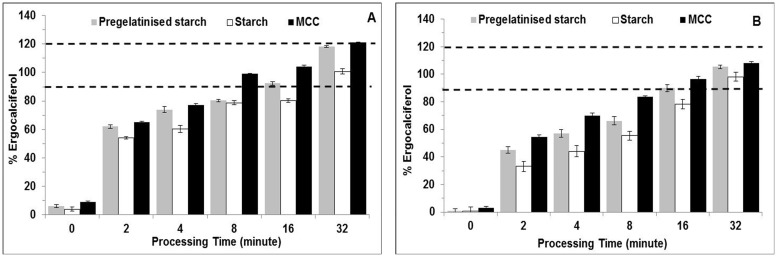
Influence of processing time and carrier type on content uniformity of ergocalciferol. Blends are made of (A) 1% and (B) 0.5% ergocalciferol and non-sieved carrier (pregelatinised starch, starch and MCC) blended using interactive blending technique using the novel dry powder coater at 300rpm (mean± SD, n = 9).

The blending process was then carried forward to investigate two further diluted batches of API (Fig 11). These blends were closer to low dose levels seen in manufactured formulations [8]. Although the 1% formulation was at an acceptable uniformity only for MCC after 8 minutes of blending (99%), the pregelatinised starch and starch formulations achieved uniformity after 16 and the full 32 minute blending period respectively. Therefore, it was clear that less time was required to get good content (8 minutes) with MCC compared to pregelatinised starch and starch. On the other hand, more time was required for starch as significantly more blend was present in the non-blending zone which was observed after blending was ceased. Thus, any API held in this zone would have a greater impact on sample uniformities obtained, and this problem is well documented with low dose blends [8]. Furthermore, a smaller API concentration would be more likely to experience segregation of API due to the comparatively reduced interactions with the larger MCC particles, as well as the tendency for API to form agglomerates in blends at concentrations below 3% [42, 43]. However, in this study, homogeneity with MCC and pregelatinised starch was achieved quicker due to the formation of ordered mixing between fine API particles and the surface of the excipient. These two excipient surfaces showed high degree of roughness which may promote interactive blend formation. The air blade in the device enables deagglomeration of the API and aids collision between particles through both convective and diffusional currents resulting in interactive blends.

## Conclusion

The development of formulation with low API content is challenging and requires a proper understanding of API and excipients properties. In this study, it was found that flow properties improved as particle size increased, with the non-sieved, non-cohesive MCC and non-cohesive pregelatinised starch having excellent flow whereas the cohesive pregelatinised starch and starch had an angle of repose just outside the threshold of being considered to have poor flow properties. In the case of geometric blending technique for one minute, all formulations failed to meet the required pharmacopeial standards for drug content uniformity. Therefore, an increase in blending time to 5 minutes with geometric addition showed considerable uniformity improvements for all carrier types. Ordered blending with the dry powder hybrid mixer allowed for uniformity to be reached faster than manual order blending, and was able to keep the blend containing cohesive powder within the acceptable uniformity range throughout the blending period. The device was also useful in obtaining good uniformities at 1% and 0.5% API using non-sieved carriers. The study concluded that MCC showed better drug content uniformity for both of the blending techniques. The results also showed the practicality and superiority of the one-step dry powder hybrid mixing device compared to geometric blending, as the non-sieved pregelatinised starch and MCC resulted in superior uniformity of content compared to geometric blending upon blending for only two minutes.
